# Estimation of Radial Basis Function Network Centers via Information Forces

**DOI:** 10.3390/e24101347

**Published:** 2022-09-23

**Authors:** Edilson Sousa Júnior, Antônio Freitas, Ricardo Rabelo, Welflen Santos

**Affiliations:** Technology Center, Universidade Federal do Piauí, Teresina 64049-550, PI, Brazil

**Keywords:** information theory, Entropy, Radial Basis Functions Networks, classification, clustering and outliers

## Abstract

The determination of The Radial Basis Function Network centers is an open problem. This work determines the cluster centers by a proposed gradient algorithm, using the information forces acting on each data point. These centers are applied to a Radial Basis Function Network for data classification. A threshold is established based on Information Potential to classify the outliers. The proposed algorithms are analysed based on databases considering the number of clusters, overlap of clusters, noise, and unbalance of cluster sizes. Combined, the threshold, and the centers determined by information forces, show good results in comparison to a similar Network with a k-means clustering algorithm.

## 1. Introduction

Broomhead and Lowe in 1988 [1] presented the Radial Basis Function Network (RBFN) concept. It is a universal approximator [2,3]. Usually, the training of an RBFN is done in two stages: initially, the centers $c_{j}$ and the variance $\sigma_{j}$ of the basis functions are determined, then, the network weights $w_{ij}$. The performance of the RBF Network depends on estimation of these parameters. This work focuses on determining the RBF centers.

Clustering techniques can be used to determine the RBF centers. These techniques find the cluster centers that reflect the distribution of the data points [4]. The most common is the k-means algorithm [5]. Other clustering techniques have been developed for RBF center identification. Examples include self-constructing clustering algorithm [6], nearest neighbor-based clustering [7] and quantum clustering [8]. Besides clustering methods, there are other techniques to estimate the RBF centers such as recursive orthogonal least squares [9] and metaheuristic optimisation [10,11].

This work proposes two algorithms that are developed mainly based on two concepts of Information Theory: the Information Potential (IP) and Information Force (IF). Those concepts describe, respectively, the amount of agglomeration and the direction where this agglomeration increases to [12]. These concepts are used in some clustering techniques, such as the one developed by Jenssen et al. [13].

The main algorithm finds the cluster centroids by a gradient ascent technique using Information Forces, and these centers are applied to an RBFN in classification problems. The second one uses the concept of Information Potential to reduce the number of outliers on noise data, and increases the performance of the RBFN. These algorithms constitute the contributions of this work.

The algorithms are tested on datasets with gradual increase in difficulty. The difficulty factors analysed are the number of clusters, overlap of clusters, noise, and unbalance of cluster sizes. The results are compared with a similar RBFN with centers estimated via k-means algorithm.

This article was organised as follows: In Section 2, the RBFN is illustrated; In Section 3, the concepts of Information Potential and Force are described, also the algorithm to estimate the RBFN centers is presented. In Section 4, the algorithm to reduce the outliers is described; In Section 5, the Data is presented and the experiment is described; In Section 6, the algorithm parameters are analysed, and the results are displayed and discussed. The conclusions are presented in Section 7.

## 2. Radial Basis Function Network

The RBFN is the function presented in the equation:

(1) $$Class_{i}=f\left(p\right)=w_{0}+\sum_{j=1}^{K}w_{ij}\varphi_{j}\left(p\right)$$

**Figure 1:**
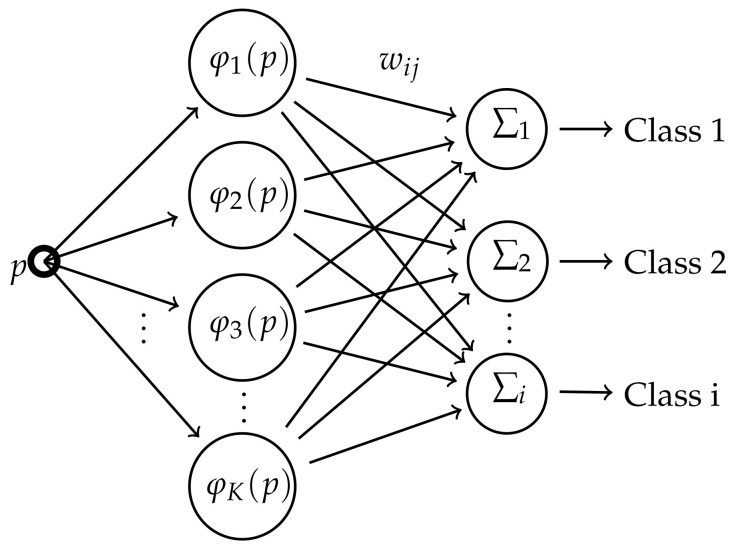
Structure of Radial Basis Function Network.

This Network is shown in Figure 1:

The RBF network is composed by an input layer *p*, a hidden layer, and an output layer which provide the classification. When an input datapoint *p* is fed into a node, the distance is calculated from a center $c_{j}$, transformed by a Radial Basis Function $\varphi_{j}\left(\cdot \right)$ and multiplied by a weighting value $w_{ij}$ [14]. All the values produced in the *K* nodes are summed for each class, and the point *p* is classified where this sum is maximum.

The methods to obtain those parameters influence the classification performance. In this work, the centers are estimated via IF and, for comparison, the k-means algorithm [15,16,17]. The weights $w_{ij}$ are determined by pseudoinverse matrix and a Gaussian Function are chosen for RBF:

(2) $$\varphi_{j}\left(p\right)=\exp\left(-\frac{||p-c_{j}{\left|\right|}^{2}}{2\cdot \sigma^{2}}\right)$$

The variance is estimated by the equation proposed by Haykin [5]:

(3) $$\sigma =\frac{d_{max}}{\sqrt{2\cdot K}}$$

In which $d_{max}$ is the maximum distance between the cluster centers and *K* is the number of nodes. The variance is equal for all nodes.

## 3. RBF Centers Estimation via IF Gradient Algorithm

### 3.1. Information Forces

Considering $x_{i}\in R^{m}$, i=1,2,…,n a set of samples belonging to a random variable $X\in R^{m}$, a Parzen Window [18] can be associated with a Gaussian kernel, directly estimating the Probability Density Function (PDF) of the data. This function can be described by:

(4) $${\hat{f}}_{x}\left(x\right)=\frac{1}{n}\sum_{i=1}^{n}G\left(x-x_{i},h^{2}\right)$$

where *G* is the Gaussian Kernel and *h* is the kernel bandwidth. There are several ways to estimate the ideal bandwidth *h* [19]. In PDF’s that are near to the Normal distribution the Rule-of-Thumb [20] is the most practical and simple one:

(5) $$h=1.06\cdot \min\left\{\hat{\sigma },\frac{IQR}{1.34}\right\}n^{-\frac{1}{5}}$$

where *n* is the number of data points, $\hat{\sigma }$ is the estimated standard deviation of the dataset, and $IQR=Q_{3}-Q_{1}$ is the interquartile range. $Q_{1}$ and $Q_{3}$ are, respectively, the first and third quartile.

The Renyi entropy equation of order two [21] is given by

(6) $$H_{R_{2}}\left(X\right)=-\log\left[\int_{X}f_{x}^{2}\left(x\right)dx\right]$$

Applying the Parzen Window:

(7) $$\begin{array}{c} \int_{X}f_{x}^{2}\left(x\right)dx=\\ \int {\left[\frac{1}{n}\sum_{i=1}^{n}G\left(x-x_{i},h^{2}\right)\right]}^{2}dx=\\ \frac{1}{n^{2}}\sum_{i=1}^{n}\sum_{j=1}^{n}\int G\left(x-x_{i},h^{2}\right)G\left(x-x_{j},h^{2}\right)dx=\\ \frac{1}{n^{2}}\sum_{i=1}^{n}\sum_{j=1}^{n}G\left(x_{i}-x_{j},2\cdot h^{2}\right) \end{array}$$

That means:

(8) $$H_{R_{2}}\left(X\right)=-\log\left[\frac{1}{n^{2}}\sum_{i=1}^{n}\sum_{j=1}^{n}G\left(x_{i}-x_{j},2h^{2}\right)\right]$$

The argument of the natural logarithm above is the Information Potential over all the dataset, in an analogy with the potential energy of physical particles [22].

(9) $$P\left(\left\{x\right\}\right)=\frac{1}{n^{2}}\sum_{i=1}^{n}\sum_{j=1}^{n}G\left(x_{i}-x_{j},2h^{2}\right)$$

The IP over a single point $x_{i}$ in the dataset is the sum of interactions of this point across all the dataset.

(10) $$P\left(\left\{x_{i}\right\}\right)=\frac{1}{n^{2}}\sum_{j=1}^{n}G\left(x_{i}-x_{j},2h^{2}\right)$$

The IP indicates the amount of agglomeration around the point. Its derivative is the Information Force acting in point $x_{i}$ [13].

(11) $$\begin{array}{c} F_{if}=\frac{\partial }{\partial (x_{i}-x_{j})}P\left(\left\{x_{i}\right\}\right)=\\ -\frac{1}{N^{2}\cdot h^{2}}\sum_{j=1}^{n}G\left(x_{i}-x_{j},2h^{2}\right)\cdot \left(x_{i}-x_{j}\right) \end{array}$$

### 3.2. Gradient Algorithm

The IF points in the direction where the amount of agglomeration increases. Then, a center candidate $c_{i}$ can approximate the central cluster by successive interaction of the equation:

(12) $$c_{i}\left(s+1\right)=c_{i}\left(s\right)+\eta F_{if}$$

This candidate $c_{i}$ could erroneously converge to a local maximum, similar to other algorithms based on Gradient Descent/Ascent. Two approaches via Information Theory could minimise this error. The first one is reducing the number of local maximum by smoothing the IP distribution over [13]. The ideal *h* (Equation (5)) is multiplied by a parameter κ which smooths or under-smooths the PDF’s distribution over.

Another solution is to variate the learning rate η over the data space. The magnitude of the IF is bigger in the border of the cluster and decreases as the candidate *c* approximates to the central cluster and the force vectors are balanced out. Then,

(13) $$\eta =\alpha \cdot e^{\left|\right|F_{\mathrm{if}}\left|\right|}$$

Outliers also hinder the IF gradient algorithm. Candidates with small Information Potential behave like outliers. Then, they are removed for the center estimation. The detailed description of the IF Gradient Algorithm is illustrated in Algorithm 1.

**Algorithm 1** Estimation of RBFN centers via IF     **Input**
  •$X_{train}$         % Data to train the RBFN;  •α         % Learning rate constant;  •n_center         % Number of initial candidates;  •δ         % Threshold of Information Potential;  •β         % Minimum distance between candidates;max_epochs         % Maximum epochs;  •γ         % Constant of convergence;     **Output**
  •$C_{cluster}$         % Cluster centers.1: **procedure** IF_algorithm($X_{train}$,α,n_center,δ,β,max_epochs,γ) 2: 3:     % $P_{if}\left(\cdot \right)$ calculates the information potential. 4:     % $F_{if}\left(\cdot \right)$ calculates the information force. 5:     **for** i∈1:n_center **do**          % Raffle the candidates 6:        $C_{cand}\left(i\right)=random\left(X_{train}\right)$; 7:         **if** $P_{if}\left(C_{cand}\left(i\right)\right)<\delta$ **then** 8:            Eliminate $C_{cand}\left(i\right)$; 9:         **end if** 10:     **end for** 11: 12:     s=0; 13:     **while** (s<max_epochs) and (not all $C_{candidate}$ are eliminated or converged) **do** 14:         % Eliminate points to close each other 15:         **for** *i* and j∈1:n_center **do** 16:            **if** $||(C_{cand}\left(i\right)-C_{cand}\left(j\right))||<\beta$ **then** 17:                Eliminate $C_{cand}\left(i\right)$ 18:            **end if** 19:         **end for** 20: 21:         **for** i∈1:n_center **do**             ▹ Update the center candidate. 22:            $C_{cand}\left(i,s+1\right)=C_{cand}\left(i,s\right)+\alpha *e^{\left|\right|F_{if}\left(i,s\right)\left|\right|}*F_{if}\left(i,s\right)$ 23:            **if** $|e^{\left|\right|F_{if}\left(i,s+1\right)\left|\right|}-e^{\left|\right|F_{if}\left(i,s\right)\left|\right|}|<\gamma$ **then** 24:                $C_{cand}\left(i\right)$ **converge!** 25:            **end if** 26:         **end for** 27:         s=s+1 28:     **end while** 29:     $C_{cluster}=\cup C_{cand}$ that converge. 30:** return** $C_{cluster}$ 31: 32:** end procedure**


Initially, a set of center candidates *c* is raffled between the dataset. This set is sufficiently big to ensure that at least one point is raffled on each cluster. Some candidates could be too close. In this case, one of the points is eliminated. Many candidates tend to converge to a single central cluster. On each interaction, if two candidates are too close to each other, one of them is eliminated.

The points raffled with small IP constitute another problem. Far from the central cluster, the greatest information force is exerted by the point initially picked. In this way, the center candidate $c_{i}$ is stuck to the starting point. To avoid that, the IP is calculated in the initial epoch over the candidates. If it is below a threshold, the center candidate is eliminated.

The interactions over a specific center candidate stop when the difference is reached:

(14) $$|\exp\left(|F_{\mathrm{if}}|\right)\left(s+1\right)-\exp\left(|F_{\mathrm{if}}|\right)\left(s\right)|<\gamma$$

where γ is a small value described in parameters section. When a center candidate nears a cluster center, the forces tend to equilibrium and the left-hand side of inequality (14) tends to zero.

The algorithm completely stops when all the candidates’ centers converge or are eliminated. If they do not converge, it stops when it reaches the maximum number of epochs.

## 4. Outlier Reduction

The RBFN has difficulties in identifying the outliers. A mechanism of outlier detection improves the RBFN results. This can be done by observing the IP on each point, because outliers have small information potential.

A threshold δ can be established with the training data. Then, this threshold can be applied to the test data and most outliers can be identified. The detailed description of this mechanism is in Algorithm 2 below.

The threshold δ is estimated using the IP values of the outliers. Some points in the clusters also have small IP and could be erroneously classified as outliers. The constant θ (<1) is established to avoid this problem.
**Algorithm 2** Outlier Detection     **Input**
  •$X_{train}$         % Data to train the RBFN;  •$X_{test}$         % Data to test the RBFN;  •n_outlier         % n° of outliers in training data;  •θ         % Constant of outlier reduction;     **Output**
  •$C_{Outlier}$         % Set of outliers.1:** procedure** Outlier_detection($X_{train}$,$X_{test}$,n_outlier,θ) 2: 3:     **for** $i\in 1:size(X_{train})$ **do** 4:         $pot_{trei}\left(i\right)=P_{if}\left(X_{train}\left(i\right)\right)$         % The IP. 5:     **end for** 6: 7:     $pot_{trei}=sort\left(pot_{trei}{,}^{\prime }ascend^{\prime }\right)$         % Sort in ascend order. 8:     $\delta =pot_{trei}\left(\theta \cdot n\_outlier\right)$         % The threshold. 9: 10:     **for** $i\in 1:size(X_{test})$ **do** 11:         **if** $P_{if}\left(X_{test}\left(i\right)\right)\leq \delta$ **then** 12:            $X_{test}\left(i\right)\in C_{Outlier}$ 13:         **end if** 14:     **end for** 15: **return** $C_{Outlier}$ 16: 17:** end procedure**


## 5. Data and Experiment

The k-means and the IF algorithm have random initialisation and the results oscillate depending on the set of initial points. Then, the algorithm is tested over different simulations and the average performance is collected. Experimentally, it was considered that one hundred simulations for each configuration is enough.

The algorithms are tested on synthetic and non-synthetic data. Each set is divided into three subsets: train, validation, and test, in a ratio respectively of 70%/15%/15%. The performance of each RBFN configuration is measured by the percentage of correctly classified points on the test set.

Synthetic data with known ground truth can lead to a best analysis of the algorithms in clustering research because the characteristics of the data can be controlled [23]. The performance of the algorithm is studied in synthetic datasets with a gradual increase of:

- Number of clusters;
- Cluster overlap;
- Unbalance in cluster size;
- Noise data.

The data come from the *Clustering basic benchmark* [23]. The characteristics of the data are presented in Table 1.

**Figure 2:**
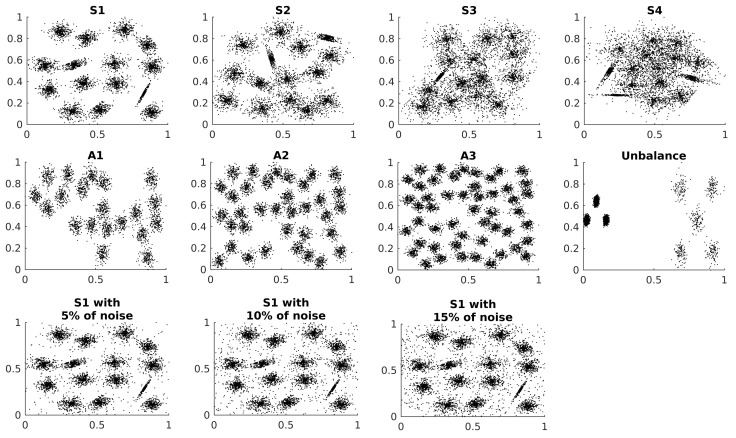
Synthetic dataset distributions.

The noise dataset is generated by the addition of independent and identically distributed random points to the first S dataset. The data distribution of the datasets presented in Table 1 are shown in Figure 2.

All synthetic datasets are two-dimensional, and the points are normalised in the interval (0,1) for each dimension. Each sequence of dataset has an ascendant level of complexity in relation to the principal characteristic. The clusters are Gaussian distributions, some of them are skewed. Also, small datasets are artificially generated via the *Multidimensional Dataset Generator for Clustering* (MDCGen) [27] to analyse the parameters of the Algorithm 1.

The *Clustering basic benchmark* also supplies the ground truth centroids for each synthetic dataset. The IF algorithm and the k-means also are evaluated on the capacity to correctly locate the estimated centroids. This evaluation is done by calculating the average distance from the estimated cluster centers and their near ground truth centroids. This measure of performance, the Average Distance to the Truth Centroid (ADTC), is presented on the equation:

(15) $$ADTC=\{\begin{array}{c} \frac{1}{n}\sum_{i=1}^{n}\min_{i,j}\left|c_{gt\;i}-c_{est\;j}\right|\;\mathrm{if}\;n\geq m\\ \frac{1}{n}\sum_{j=1}^{n}\min_{i,j}|c_{gt\;i}-c_{est\;j}|+\frac{1}{m-n}\sum_{j^{\prime }=1}^{m-n}\min_{j^{\prime },i}\left|c_{est\;j^{\prime }}-c_{gt\;i}\right|\;\mathrm{with}\;j\neq j^{\prime }\;\mathrm{if}\;n<m \end{array}$$

where $c_{est}$ and $c_{gt}$ are, respectively, the estimated and the ground truth centroids, with *n* and *m* as their respective number of elements. Different from the k-means, IF algorithm does not have the number centroids as parameter and can mistake the real number of clusters and their centroids. The Equation (15) also penalises when the estimated number of centroids is different from the number of clusters.

Non-synthetic datasets are also important to evaluate the algorithm performance on real problems. The Iris Dataset [28,29] is used to analyze the algorithms. This database is one of the best known to be found in the pattern recognition literature.

## 6. Parameters and Results

### 6.1. Parameters

#### 6.1.1. Learning Rate Constant

**Figure 3:**
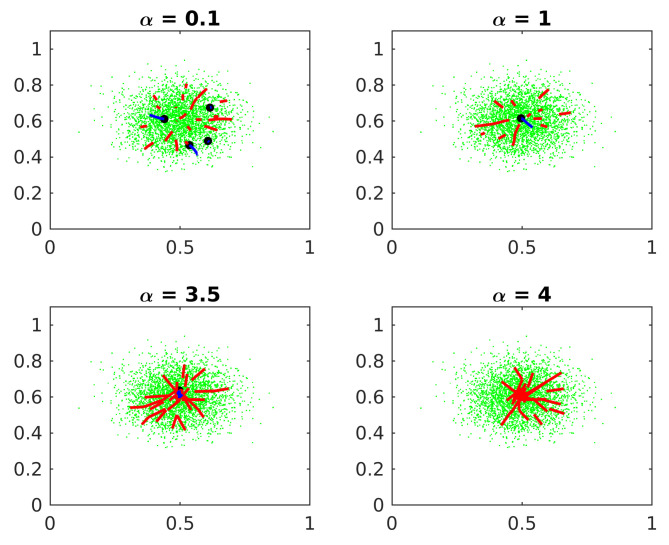
Learning rate constant. The black dots represent the estimated cluster centers. The red lines represent the trajectory of the eliminated candidates. The blue lines represent the converged ones.

Figure 3 shows the effect of the Learning rate constant α on Algorithm 1.

If the constant α is too small, some centers candidates erroneously converge to local maxima. If α is too big, the candidates oscillate around the central cluster and the algorithm loses accuracy. If α is very big, the algorithm does not converge before reaching the maximum epochs. Experimentally, a good value for α is ten times the standard deviation of the clusters.

#### 6.1.2. Minimum Distance between Centers Candidates

**Figure 4:**
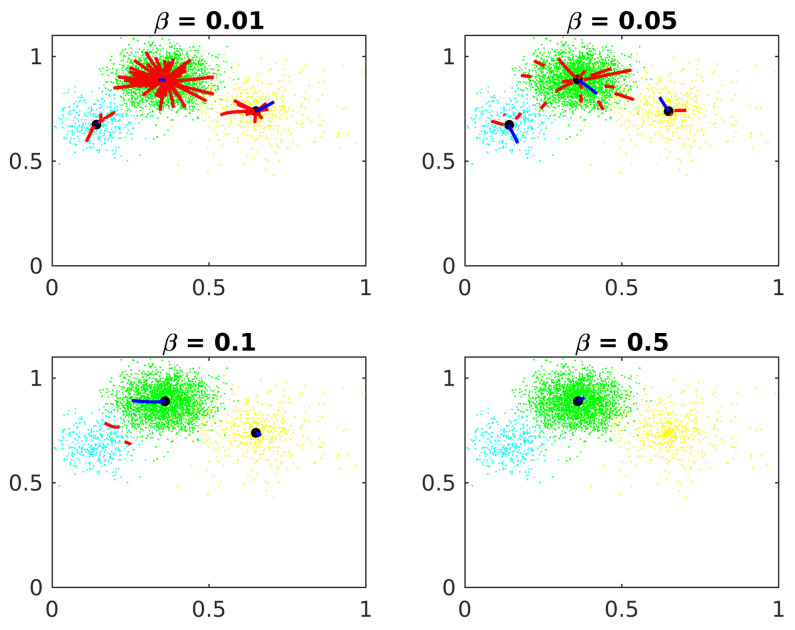
Minimum distance between centers candidates.

Figure 4 shows the constant β effects on Algorithm 1.

If this distance is too small, just a few points are eliminated on each epoch and the algorithm demands more computational effort. If β is too big, a center candidate in one cluster could eliminate good center candidates in other clusters. Experimentally, a good value for β is 10% of the standard deviation of the clusters.

#### 6.1.3. Constant of Convergence

**Figure 5:**
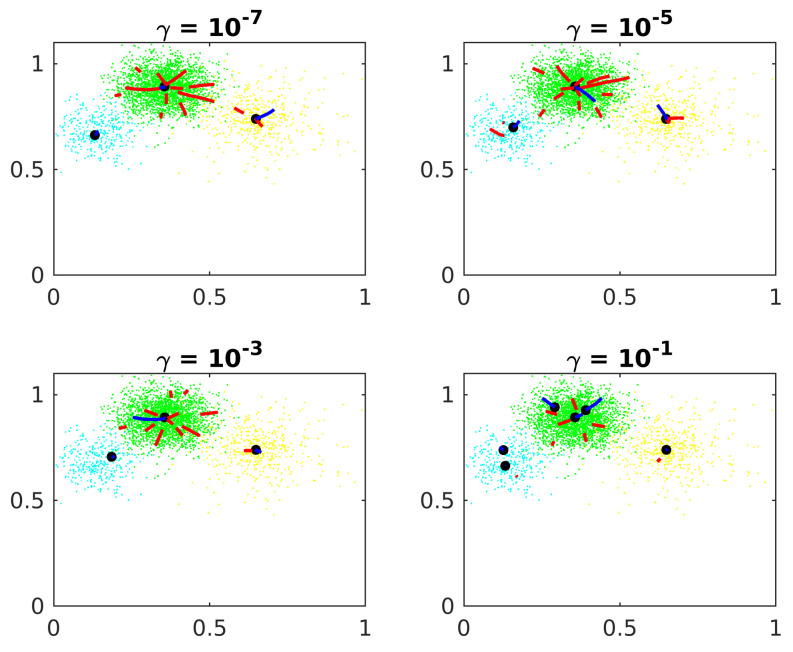
Constant of convergence.

Figure 5 shows the constant γ effects on Algorithm 1.

The precision increases when the constant γ diminishes, although the algorithm takes more epochs to converge, requiring more computational effort. If γ is too big, the center candidates stop before the forces balance out, far from the actual central cluster. Experimentally, $\gamma =10^{-6}$ is an appropriate value.

#### 6.1.4. IP Threshold

**Figure 6:**
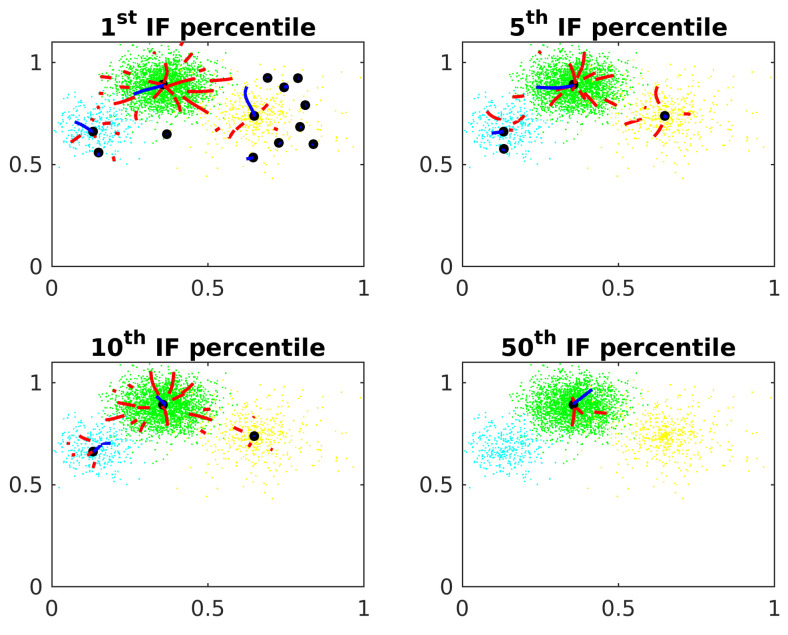
IP threshold.

Figure 6 shows the threshold δ effects on the Algorithm 1. The potential is calculated at each point. The threshold is tested as the 1st, 5th, 10th and 50th percentile from the IP distribution of the points.

Some center candidates are raffled on points with small IP. These candidates are stuck close to the origin, not converging to the actual cluster centers. If the threshold δ is too small, these center candidates are not eliminated by the algorithm. In the other side, if δ is too big, it eliminates good center candidates in clusters with small IP.

#### 6.1.5. Smoothie Parameter

**Figure 7:**
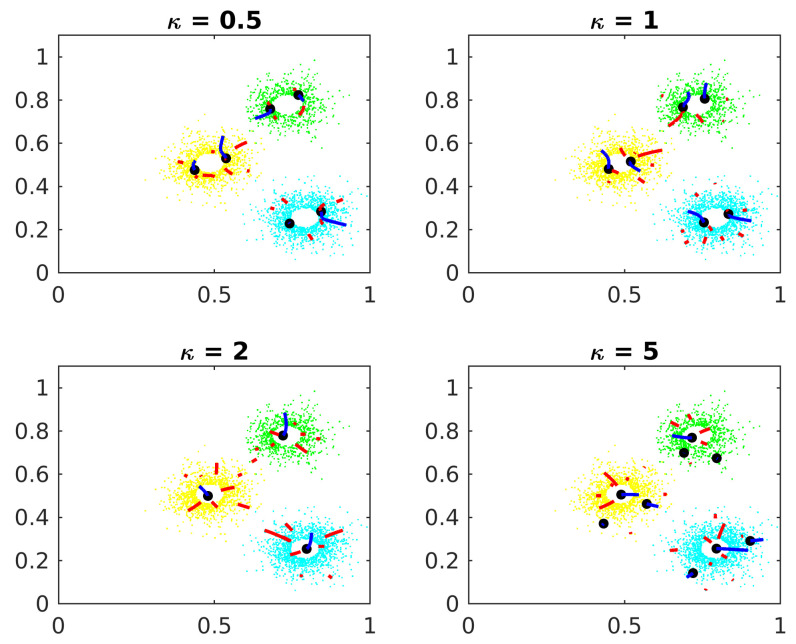
Smoothie parameter.

Figure 7 shows the parameter κ effects on Algorithm 1.

If κ is small, the candidates converge to local maxima inside the clusters but far from the actual center. If κ is big, points too far from the central cluster exert too much influence in the IF vectors, confusing the gradient algorithm.

#### 6.1.6. Constant of Outlier Reduction

Table 2 shows, in dataset S1 with 10% of noise, the performance of the RBFN associated with Algorithm 2 and percentage of the points correctly classified as outliers using different values of the parameter θ.

As described in Section 2, some points inside the cluster but far from the actual center have small IP. The parameter θ partially avoids that the outlier reduction algorithm misclassifies these points. The value of θ depends on the concentration level of points in the clusters.

### 6.2. Results

#### 6.2.1. Number of Clusters

**Figure 8:**
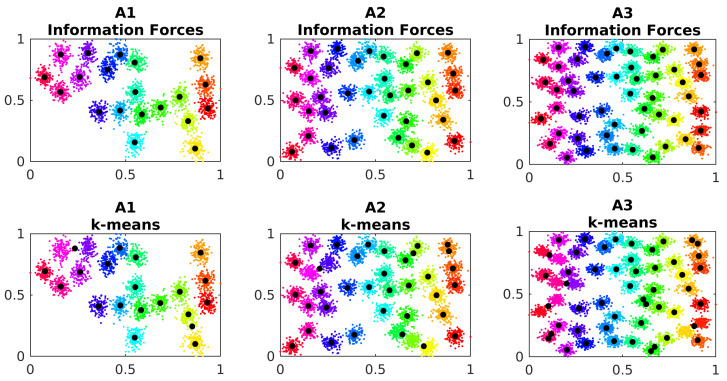
Estimated cluster centers in dataset A. The black dots represent the estimated cluster centers.

The RBFN centers are estimated by information forces (Algorithm 1) and by k-means algorithm for comparison. The A datasets are used to analyse the effects of the number of clusters in the performance of the algorithms. The κ parameter from the IF algorithm is kept around 0.2 in order to under-smooth the data distribution, and, consequently, the algorithm can better differentiate the clusters. The Figure 8 show the centers location estimated for each method on each A dataset.

**Figure 9:**
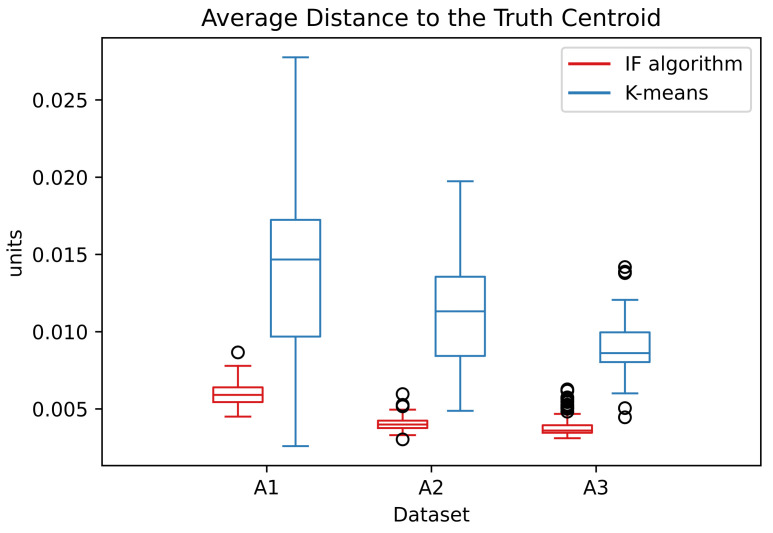
Distribution of the ADTC values in dataset A over the simulations.

The k-means algorithm presents difficulties in correctly estimating the centroids, as it can be observed in Figure 8. The information forces point to one center on each cluster, closer to their centroids. The Table 3 shows the average distance between the estimated centers and their near ground truth centroids for the simulations:

The ADTC measure for the IF algorithm is smaller than for the K-means. This indicate that the centers estimated by the IF algorithm are nearer from the ground truth centroids than the estimated ones by the k-means. The ADTC values presented in Table 3 are the average over the repeated simulations. The distribution of the ADTC values are presented in the Figure 9:

In the A datasets, the ADTC values from the IF algorithm are distributed in a small interval. This distribution is smaller than the correspondent k-means. This indicates that the IF algorithm has a better capacity in converge to the correct centroids. The estimated centers are applied to the RBFN. The percentage of correctly classified points are presented in Table 4.

The results of the RBFN with centers estimated via Information Forces are similar to the analogue RBFN with centers estimated via k-means. However, the IF algorithm can handle the increasing number of clusters and better locate the RBF center, which supplies more stable results.

#### 6.2.2. Cluster Overlap

**Figure 10:**
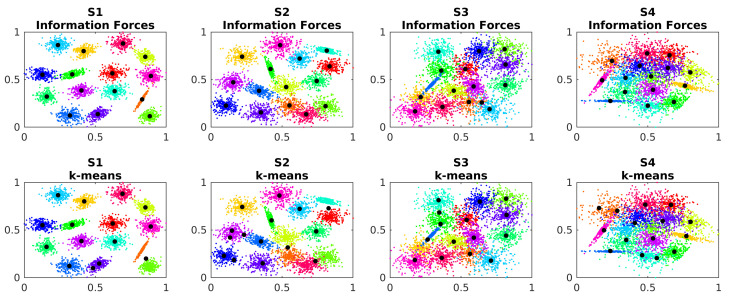
Estimated cluster centers in dataset S.

The S datasets are used to analyse the effects of the overlap between the clusters in the performance of the algorithms. The Figure 10 shows the centers’ estimated location for each method on each S dataset.

Similar to the A dataset, the k-means also has difficulties in correctly locate the centroids and the IF algorithm better estimate them. The Table 5 shows the ADTC measure for the S dataset.

**Figure 11:**
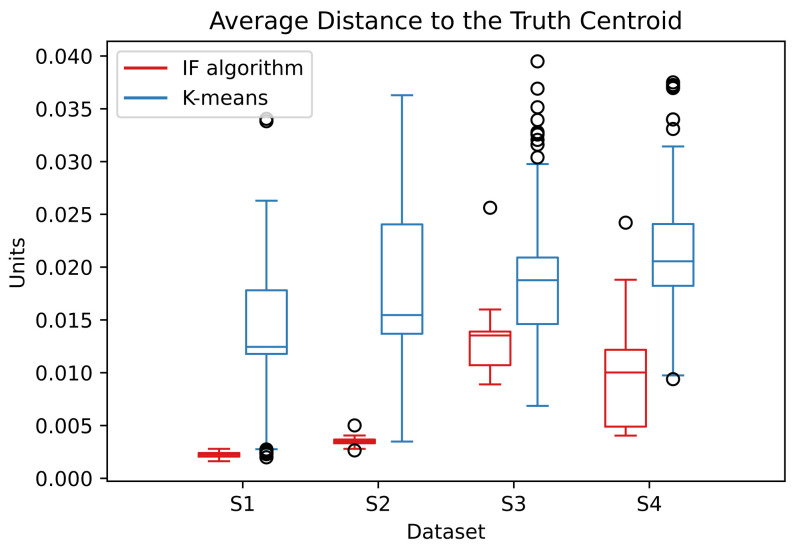
Distribution of the ADTC values in dataset S over the simulations.

The IF algorithm can better handle the cluster overlap and locate the centroids closer to the ground truth. The κ parameter must also under-smooth the data distribution for the algorithm better differentiate the clusters. The distribution of the ADTC values are presented on the Figure 11:

The ADTC values for the IF algorithm stay in a smaller interval which indicates a better convergence. The percentage of correctly classified points by the RBFN are presented in Table 6.

Analogue to the dataset A, the IF algorithm gives similar results to the k-means’ in the classification of the out-of-sample data. However, the RBF centers estimated by IF algorithm are better located, which gives more robust results.

#### 6.2.3. Unbalance in Cluster Size

Clustering algorithms with random initialization have difficulties to handle datasets where the clusters have big differences in number of points. The probability to sort the right amount of points on each cluster tend to diminish when the unbalancing in the number of points in the clusters tend to increase. The k-means algorithm has this weakness [23].

**Figure 12:**
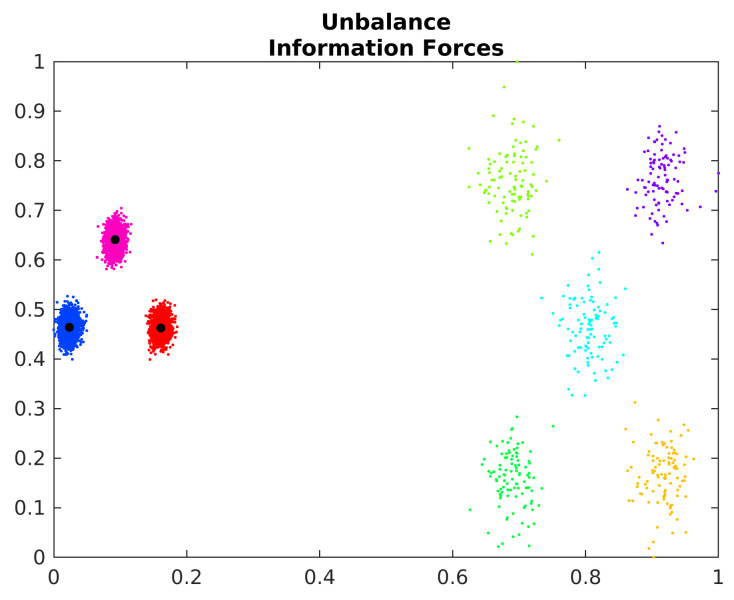
Estimated cluster centers in the Unbalance dataset.

The IF algorithm also has random initialization, and, consequentially, has difficulties in unbalance clusters. Figure 12 shows the estimated cluster centers by the IF algorithm on the Unbalance dataset.

The IF algorithm incorrectly identifies the points of the less dense clusters as outliers. However, the centroids of denser areas are correctly estimated. In this way, the algorithm can be used with other strategies to identify the denser areas and better estimate the centroids of less dense clusters.

#### 6.2.4. Noise Data

**Figure 13:**
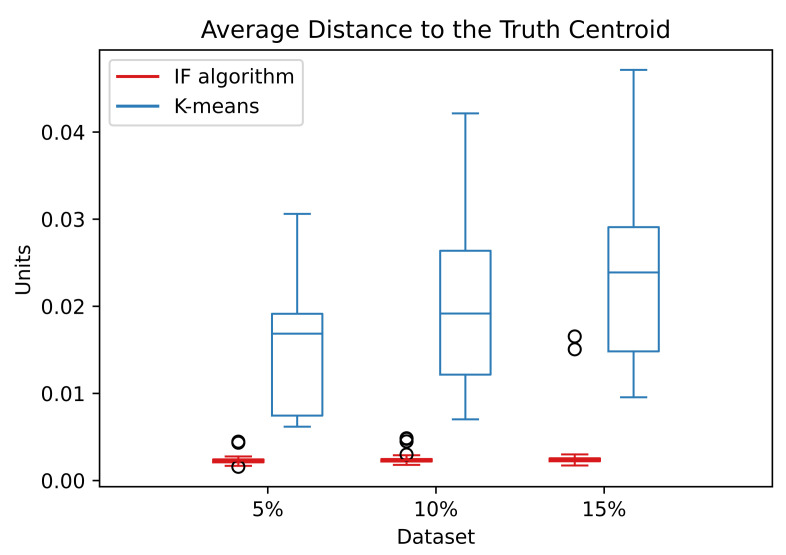
Distribution of the ADTC values in the noise dataset over the simulations. The abscissa refers to the percentage of random noise added to the S1 dataset.

The Figure 13 shows the distribution of the ADTC values for the noise data:

The ADTC values for the IF algorithm are smaller and located at a small interval than the correspondent k-means. This indicates that the IF algorithm better estimates the centroids with a better convergence. The information forces exerted by the random noise points have the tendency to balance out and does not disturb the IF algorithm. Even with the increase of random noise in the data, the ADTC values stay very similar.

The Table 7 shows the performance of the RBFN on noise data. The centers are estimated via k-means and the IF algorithm, with and without the noise reduction by the Algorithm 2.

The IF gradient algorithm without outlier reduction has a reasonable performance on this dataset. There is an improvement when the outlier reduction is used alongside the RBFN with centers estimated by IF. This improvement leads the IF gradient algorithm to outperform the k-means.

#### 6.2.5. Iris Dataset

**Figure 14:**
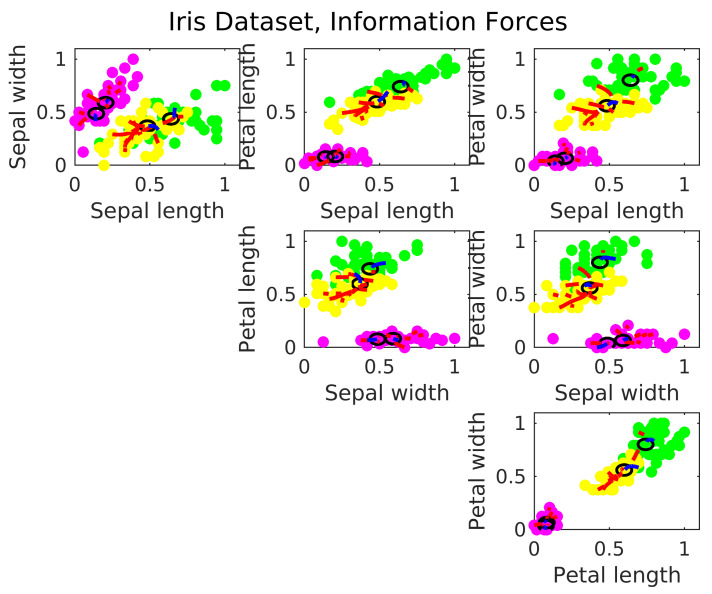
Estimated cluster centers in the Iris dataset via IF.

The Iris Data is a four dimension non-synthetic dataset. It is formed by three classes, each one referring to a type of iris plant. One of the clusters is linearly separable from the other two, however, the other two are not linearly separable from each other. The Figure 14 shows the centers location estimated via IF.

The clusters in the Iris data are skewed and not radial. The IF algorithm estimate the centers following the level of agglomeration in the data. Then, there is the tendency in the Iris Data to estimate more than one center per cluster, and locate them far from the geometrical center. With the IF algorithm, the RBFN has a accuracy of 90.87%, against 89.74% from a similar neural network operating with the k-means.

#### 6.2.6. Discussion and Future Works

The proposed algorithm constitutes a tool that, in comparison with k-means, has a good ability to identify cluster centroids in datasets, centroids which are used as centers of RBF network. The IF algorithm can handle the increase in the number of clusters and cluster overlap. On Noise Data, the outlier reduction improves the RBFN results. The algorithm demonstrates some difficulties on the Unbalance dataset, however, the results may still lead to solutions for handle this characteristic on data.

The preliminary results show good accuracy of the RBFN configured with the IF algorithm. Other studies may analyze the performance of the proposed methods on more complex databases. Further on, it may analyze how the RBFN behaves with the IF gradient algorithm alongside other methods to determine the basis function variance and the network weights.

The ability of the IF algorithm to estimate the cluster centroids may also improve clustering algorithms. Future works may use the IF algorithm ability to search cluster centroids as an initialization technique for clustering algorithms, replacing the random initialization present in some clustering techniques such as the k-means itself. Also, the IF algorithm may be used in association with density based clustering techniques to find denser areas in the data.

## 7. Conclusions

This proposed method to assign the RBFN centers presents satisfactory preliminary results in comparison to the traditional k-means algorithm. Also, the outlier reduction based on information potential improves the results on noise data. It is noteworthy that the proposed method accuracy depends on the correct adjustment of some parameters, but this also happens in other methods.

## Figures and Tables

**Table 1:** Datasets characteristics.

Dataset	Varying	Size	#Clusters	Per Cluster	%Outliers	Source
S	Overlap	5000	15	333	0	[24]
A	Number of clusters	3000–7500	20;30;50	150	0	[25]
Unbalance	Balance in cluster size	6500	8	100–2000	0	[26]
Noise	Outliers	5250–5750	15	150	5%;10%;15%	Authors ^1^

^1^ The S dataset with random noise.

**Table 2:** Accuracy of the RBFN associated with the outlier reduction for different θ values.

θ	Points Correctly Classified	Outliers Correctly Classified
0.5	93.69%	39.34%
0.75	94.06%	54.10%
1	93.21%	63.93%

**Table 3:** Average Distance to the Truth Centroid for the A dataset.

Dataset	Information Forces	k-Means
A1	0.00593	0.01396
A2	0.004053	0.01107
A3	0.003918	0.008813

**Table 4:** Percentage of Accuracy in out-of-sample data for the A dataset.

Dataset	Information Forces	k-Means
A1	95.69%	95.95%
A2	97.47%	97.49%
A3	97.54%	97.55%

**Table 5:** Average Distance to the Truth Centroid for the S dataset.

Dataset	Information Forces	k-Means
S1	0.002239	0.01359
S2	0.003487	0.01629
S3	0.01275	0.01872
S4	0.009276	0.02091

**Table 6:** Percentage of Accuracy in out-of-sample data for the S dataset.

Dataset	Information Forces	k-Means
S1	98.81%	98.87%
S2	95.73%	95.65%
S3	84.87%	84.81%
S4	80.10%	79.93%

**Table 7:** Percentage of Accuracy in out-of-sample data for the S dataset.

Dataset	Information Forces with Outlier Reduction	Information Forces without Outlier Reduction	k-Means
S1 with 5% of noise	95.05%	94.32%	94.40%
S1 with 10% of noise	91.95%	90.36%	90.50%
S1 with 15% of noise	90.68%	86.82%	87.06%

## Data Availability

The algorithms developed in this work were implemented in Matlab software and they are available at https://www.mathworks.com/matlabcentral/fileexchange/115065-if_algorithm (accessed on 8 September 2022).

## Abbreviations

The following abbreviations are used in this manuscript:

ADTC	Average Distance to the Truth Centroid
IF	Information Force
IP	Information Potential
MDPI	Multidisciplinary Digital Publishing Institute
PDF	Probability Density Function
RBF	Radial Basis Function
RBFN	Radial Basis Function Network
List of variables	
*c*	Centroids/RBF centers
$c_{est}$	estimated centroids
$c_{gt}$	ground truth centroids
$d_{max}$	Maximum distance between the cluster centers
*f*	Function
$F_{if}$	Information Force
*G*	Gaussian Kernel
*h*	Kernel bandwidth
*H*	Entropy
IQR	Interquartile range
*K*	Neural network nodes
*n*	Number of samples
*p*	Datapoint
P(·)	Information Potential
$Q_{1}$	First quartile
$Q_{3}$	Third quartile
*s*	Epoch
*w*	Weights
*x*	Sample
*X*	Set of samples
α	Learning rate constant
β	Minimum distance between candidates
γ	Constant of convergence
δ	Threshold of Information Potential
η	Learning rate
θ	Constant of outlier reduction
κ	Smoothie parameter
$\hat{\sigma }$	Estimated standard deviation
$\sigma^{2}$	Variance/RBF width
φ	Radial Basis Function
